# Altered Metabolism and Persistent Starvation Behaviors Caused by Reduced AMPK Function in *Drosophila*

**DOI:** 10.1371/journal.pone.0012799

**Published:** 2010-09-20

**Authors:** Erik C. Johnson, Nevzat Kazgan, Colin A. Bretz, Lawrence J. Forsberg, Clare E. Hector, Ryan J. Worthen, Rob Onyenwoke, Jay E. Brenman

**Affiliations:** 1 Department of Biology, Wake Forest University, Winston-Salem, North Carolina, United States of America; 2 Translational Science Center, Wake Forest University, Winston-Salem, North Carolina, United States of America; 3 Neuroscience Center, University of North Carolina, Chapel Hill, North Carolina, United States of America; 4 Department of Cell and Developmental Biology, University of North Carolina, Chapel Hill, North Carolina, United States of America; VIB, Belgium

## Abstract

Organisms must utilize multiple mechanisms to maintain energetic homeostasis in the face of limited nutrient availability. One mechanism involves activation of the heterotrimeric AMP-activated protein kinase (AMPK), a cell-autonomous sensor to energetic changes regulated by ATP to AMP ratios. We examined the phenotypic consequences of reduced AMPK function, both through RNAi knockdown of the gamma subunit (AMPKγ) and through expression of a dominant negative alpha (AMPKα) variant in *Drosophila melanogaster*. Reduced AMPK signaling leads to hypersensitivity to starvation conditions as measured by lifespan and locomotor activity. Locomotor levels in flies with reduced AMPK function were lower during unstressed conditions, but starvation-induced hyperactivity, an adaptive response to encourage foraging, was significantly higher than in wild type. Unexpectedly, total dietary intake was greater in animals with reduced AMPK function yet total triglyceride levels were lower. AMPK mutant animals displayed starvation-like lipid accumulation patterns in metabolically key liver-like cells, oenocytes, even under fed conditions, consistent with a persistent starved state. Measurements of O_2_ consumption reveal that metabolic rates are greater in animals with reduced AMPK function. Lastly, rapamycin treatment tempers the starvation sensitivity and lethality associated with reduced AMPK function. Collectively, these results are consistent with models that AMPK shifts energy usage away from expenditures into a conservation mode during nutrient-limited conditions at a cellular level. The highly conserved AMPK subunits throughout the Metazoa, suggest such findings may provide significant insight for pharmaceutical strategies to manipulate AMPK function in humans.

## Introduction

The precise mechanisms of how organisms maintain energetic homeostasis in light of dynamic food availability remains largely unknown. A molecule thought to represent a central cellular mechanism mediating energy allocation is the AMP-activated protein kinase, by virtue of its activation by the end product of ATP hydrolysis [1]. Activation of AMPK by AMP leads to the phosphorylation of many targets, including Acetyl CoA carboxylase (ACC) and the Peroxisome Proliferator Activated Receptor (PPAR), which has the net biological effect of diverting energy away from cellular processes that require energy and towards a conservation or energy production mode [2]. The functional AMP-activated protein kinase (AMPK) is a heterotrimer consisting of a catalytic alpha (α), a regulatory gamma (γ), and a scaffolding beta subunit (β) [3]. In mammals, multiple genes encode each of the subunits, which form different heterotrimeric complexes [4], so despite intense scrutiny of the roles of AMPK in mammals, the complete organismal context of AMPK function remains poorly understood. In *Drosophila*, a single gene encodes each subunit, and clear homologs of each subunit are present and display 60%, 62%, and 62% identity with human α, β, and γ subunits, respectively. *Drosophila* AMPK is highly similar to mammalian AMPK, as it is formed via a heterotrimeric complex, is activated by AMP, and has many of the same targets, including acetyl-CoA carboxylase (ACC) [5].

Genetic modulation of AMPK activity in various model systems suggests that AMPK may represent an underlying mechanism to increase longevity as a consequence of dietary restriction [6], and also that AMPK activity may be responsible for the benefits of exercise [7]. Additionally, pharmacological agents targeting AMPK activity have been developed as therapeutics for treatment of human pathologies associated with diabetes [8].

We report here that attenuated AMPK function in *Drosophila* leads to a series of behavioral and metabolic phenotypes demonstrating that these animals behave as though they are experiencing chronic starvation. Specifically, animals deficient in AMPK signaling show a hypersensitivity to starvation conditions, altered activity levels, and differential lipid quantities and distributions. These phenotypes further demonstrate a pivotal role of the AMPK molecule in the organization of behavioral and physiological responses to nutrient-limitations, and that such responses appear to be highly conserved throughout the Metazoa.

## Methods

### *Drosophila* cultures and stocks

The AMPK*^K57A^* transgene was generated by a mutagenic PCR strategy and cloned into the UAS-vector for embryo transformation. The UAS-AMPK-α WT transgene was previously described [9]. Other lines that were employed in this study were Ubi-GAL4, Act5C-GAL4, hsp70-GAL4, 109 (80) GAL4, UAS-alpha AMPK RNAi, UAS-gamma AMPK RNAi, UAS-LSD2-GFP, UAS-TOR^TED^ and the UAS-dcr 2. The UAS-dcr2 and UAS-LSD2-GFP lines were kind contributions from Dr. Paul Taghert and Ronald Kuhnlein respectively. All other lines, with the exception of the Ubiq-GAL4 and AMPK alpha transgenes were procured from the Bloomington Stock center (Bloomington, IN). All flies were maintained in an incubator maintained at 25°C and under a 12∶12 LD cycle. Flies were cultured on a standard molasses-malt-cornmeal-agar-yeast medium and housed in uncrowded conditions. All transgenes were backcrossed to the *w^1118^* background. For hsp70-GAL4 lines, we raised and maintained animals at 19°C for all assays (uninduced), and induction was carried out by placing animals at 30°C for 1 hr twice daily for three days, and then assaying animals at 25°C.

### Lifespan Measurements

Measurements of lifespan have been widely employed in *Drosophila* as a metric of stress sensitivity *e.g* [10]–[11]. We placed thirty 3 to 5 day old mated flies (males and females housed separately) in vials with a two percent agar solution to starve the animals. We assessed percent survival of at least three replicate vials twice daily. For each vial, we assessed the median survival for the treatment and data were pooled to estimate a mean median survival and then employed a one-way ANOVA with post-hoc Tukey's comparison for differences between genotypes/treatments.

### Locomotor measurements

Locomotor activity was monitored with a TriKinetics Locomotor Monitor (Waltham, MA) on the aggregate population of thirty 3–5 day old flies [12]. Flies were housed in a 12∶12 LD cycle for three days prior to the experiments. Flies were transferred to a vial containing starvation or normal medium at ZT0. Total beam counts were monitored continuously through an automated system for forty eight hours. For analysis of the starvation-induced component, we chose to examine only the first 12 hours of locomotor activity, to avoid measurements of different numbers of flies caused by differential mortality. We determined the amount of activity during starvation relative to the activity of fed conditions for the same time period.

### Transcript analysis

Ninety adult flies were homogenized in 1 mL of TriZol (Invitrogen, Ca) and total RNA was extracted according to manufacturer's recommendations. RNA was then used for cDNA synthesis using a SuperScript III Reverse Transcription Kit (Invitrogen). Specific primers for the gamma AMPK subunit ORF were designed to flank intervening sequences to distinguish if amplicons originated from cDNA versus contaminating genomic DNA. The primers for the gamma subunit were: Forward: 5′- AAAAACAAAACCAAAAGCAACAA and Reverse: 5′AATTATTGGAATTGGAGCTGGAG. Specific primers were also designed to amplify the housekeeping gene, *RP49* [12]. PCR conditions were 94° for 5 minutes, followed by 25 cycles of 94°C for 45°C seconds, 55°C for 45 seconds, and 72°C for 90 seconds, followed by a single extension of 72°C for ten minutes.

### Triglyceride and Oil Red O Measurements

Total triglyceride levels were assessed through use of a Serum Total Triglyceride Kit (Sigma, St. Louis) according to manufacturer's instructions and as previously described [12]. Thirty flies were weighed, and homogenized completely in 0.5 mL 100 mM Tris-HCl (pH 7.4), 1 NaCl, 1% TritonX-100 buffer. Homogenates were centrifuged for 2 minutes at room temperature at 13000×g to collect particulates. One hundred microliters of supernatant was transferred to a 96 well plate and an equal volume of working triglyceride reagent was added to each well. Triplicate wells were performed for each sample and three replicate samples of flies were run for each treatment condition. Absorbance at 510 nm wavelength was measured on a Perkin Elmer Victor III multilabel microplate reader. Absorbance values were compared to standards and normalized by fly weight. A homogeneity of slopes test was performed following linear regression on normalized triglyceride levels to compare different genotypes following starvation conditions. Oil Red O staining was done as described [13]. Briefly, wild-type and AMPK alpha null larvae were dissected in 4% paraformaldehyde in PBS to expose the oenocytes, then fixed for 10 minutes. After rinsing twice with water, lipid droplets were stained with a 0.06% Oil Red O solution in 60% isopropanol for 30 minutes at room temperature, and then rinsed three times with water. Stained larvae were then mounted in glycerol and visualized on a light microscope.

### Feeding Assays

To assess food intake, we employed the CAFE assay [14], which quantifies dietary intake using a volumetric capillary feeder. We placed three 3–5 day old adult flies in the CAFE chamber and monitored the dietary intake of either 5% sucrose/5% yeast extract or 5% sucrose media. Red food coloring was added to each diet to facilitate measurements. Mineral oil was placed over the top of the capillary to reduce evaporation. Animals were placed in a 12∶12 LD cycle at ZT0 (lights on). Humidity was controlled by placing the chamber over a water source. Animals were then transferred the following day and capillary tubes were marked and measured. We also determined the amount of diet that was lost to evaporation by placing a capillary feeder in a chamber without flies and this value was subtracted from the values determined for the feeding experiments. Males and females were housed in separate chambers. Differences in dietary intake were assessed using a Two-Way ANOVA. A complementary group of experiments employed variations in a two-choice assay [15]. This assay was developed to assess aversion to various stimuli and relies on scoring of an impregnated dye in the food and subsequent scoring of abdominal color. High numbers of scored individuals are predicated on a period of starvation, so we modified the number of scored individuals on a single dye in the food with variations in food access.

For rapamycin (LC Laboratories, Woburn MA) feeding, 200 adult *Drosophila* were put in cages over standard cornmeal-yeast media containing vehicle (ethanol) or 1 µM rapamycin to lay eggs overnight. Larvae were scored (n>200 for all conditions) on days 5 and 7 after egg laying and scored for the absence or presence of the green fluorescence of the FM7i balancer. Mutant survival percentages were normalized to their survival at 5 days without rapamycin (but vehicle alone). For adult rapamycin feeding, adult animals were placed on instant Carolina medium containing 100 µM rapamycin for three days prior to starvation assays.

### Oxygen Consumption measurements

A calibrated 50 µL micropipette was flame bent to fashion a micromanometer. Whatman filter paper was saturated in a KOH solution, to act as a CO_2_ scavenger, and placed in the bottom of a 15 mL polypropylene tube. Loose cotton was placed over the KOH-saturated filter paper, and 10 flies were loaded into the chamber which was then sealed with wax. Water was delivered to the end of the micromanometer and given ten minutes to equalize. After this time period, the position of the meniscus was marked and re-marked following a ten minute period for three replicates of ten minutes to estimate an average reading. Measurements were taken on two genotypes differing in AMPK function at the same time, since manometric readings may be influenced by external factors such as changes in barometric pressures, and these grouped readings were repeated six times were analyzed for statistical significance using a nested ANOVA (GraphPad, San Diego).

## Results

### Altered AMPK function causes abbreviated lifespan during starvation

To investigate the roles of AMPK in the maintenance of metabolic homeostasis in adults, we relied on two experimental strategies to alter AMPK activity. The first approach was to generate a dominant negative construct (K57A), in which the catalytic domain of the alpha subunit was altered to inactivate the ATP binding domain, as suggested by a number of studies in mammalian systems [16]–[17]. Notably, this lysine residue is present in all protein kinases and numerous studies have previously shown that this residue is absolutely required for kinase function *e.g*. [18]–[20]. As a complementary approach, we genetically introduced a double-stranded RNA species targeting the gamma subunit (γRNAi). A null mutation in the alpha subunit of AMPK causes lethality in late larval stages [9], making it necessary to adopt these experimental strategies to investigate AMPK function in adults.

To first evaluate whether the K57A variant was behaving as a loss of function allele, we tested whether expression of the K57A variant would phenocopy the lethality of the null mutant. Expression of the K57A variant with either the Act5C-GAL4 or tub-GAL4 driver completely phenocopies the lethality associated with the null mutant, as did specific RNAi constructs targeting either the alpha or gamma subunits (Figure S1). Thus, employing these ubiquitous drivers to express elements aimed at reducing AMPK expression or function results in a complete phenocopy of the lethality caused by a null mutant. Additionally, repression of GAL4-mediated transcription *via* the GAL80 element rescued the lethality associated with expression of either the RNAi or the K57A alpha variant. Next, we tested whether it could rescue the lethality caused by a null mutation in the alpha subunit [9]. Introducti of a wild-type copy of the alpha subunit in an *ampk* mutant background provided complete restoration of Mendelian segregation ratios (Figure S2). In contrast, introduction of the K57A variant failed to provide any rescue. Furthermore, co-expression of a wild-type alpha subunit completely rescued the lethality caused by overexpression of the K57A variant.

**Figure 1:**
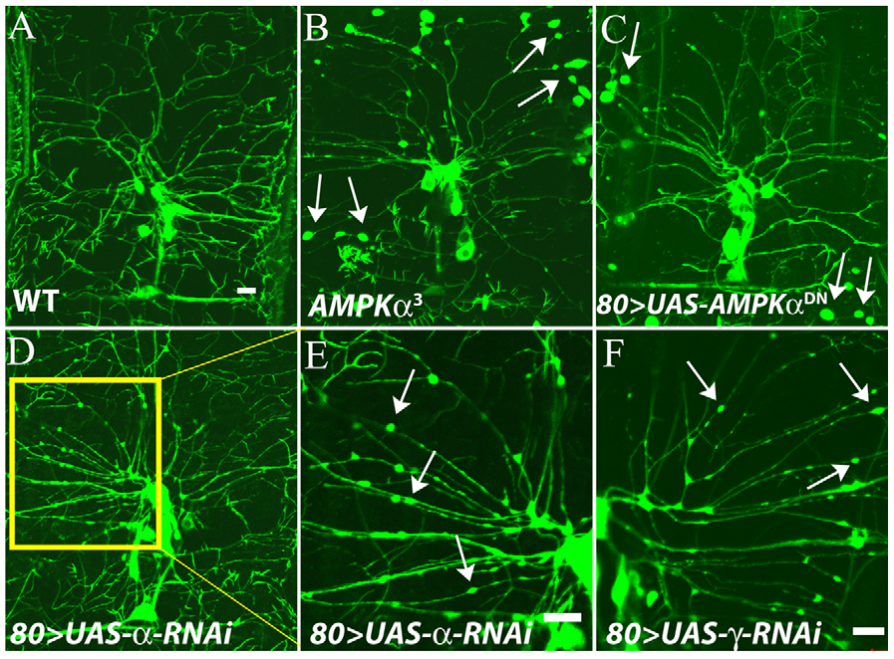
Expressing either dominant negative AMPKα or RNAi targeting αor γ subunits phenocopies a null allele of the AMPK α subunit. Representative confocal images from animals specifically expressing an actin-GFP construct in class IV multi-dendritic sensory neurons in larval *Drosophila*, with different levels of AMPK function. (A) Normal dendritic morphology of animals with wild-type AMPK. (B) Dendritic morphology in animals with an AMPKα subunit null mutation (8): note the large varicosities on neuronal dendrites. (C) Animals expressing the dominant negative alpha subunit variant which phenocopies the null mutant, showing the same abnormal varicosities (white arrows). (Scale bar = 10 µm) (D) Animals expressing an RNAi targeting the alpha subunit also phenocopies the null mutant, but with smaller varicosities (E) Inset of D. F. Animals expressing RNAi targeting the gamma subunit also, but more weakly, phenocopies the null mutant (A–E) 22°C (F) 29°C.

In early larval stages, the null mutant causes aberrant morphology of the Class IV multi-dendritic neurons [9]. Using the 109(2)80-GAL4 driver, we specifically expressed the K57A alpha variant in these sensory neurons, which results in similar morphological defects (Figure 1A-F), indicating that this element is effective at genetically reducing AMPK function in a manner similar to loss of function mutations. Consequently, we refer to this variant as the AMPK^DN^ for its dominant negative function. In parallel, we evaluated expression of the AMPKα subunit in animals expressing either an RNAi targeting the γ (γ RNAi) or α (αRNAi) subunits, and observed a significant reduction in the α protein levels in these two genotypes but not in animals possessing the GAL4 alone or in animals expressing an RNAi targeting a different gene (Figure S3). Since functional AMPK is an obligate heterotrimer, reduced levels of one subunit has previously been shown to lead to the instability of the others [21], which is consistent with our results, and suggests complex feedback mechanisms to regulate the dosage of all subunits. Likewise, direct measurement of gamma transcript levels were significantly reduced in animals expressing the gamma RNAi element as compared to animals with wild-type AMPK function (Figure S3)

**Figure 2:**
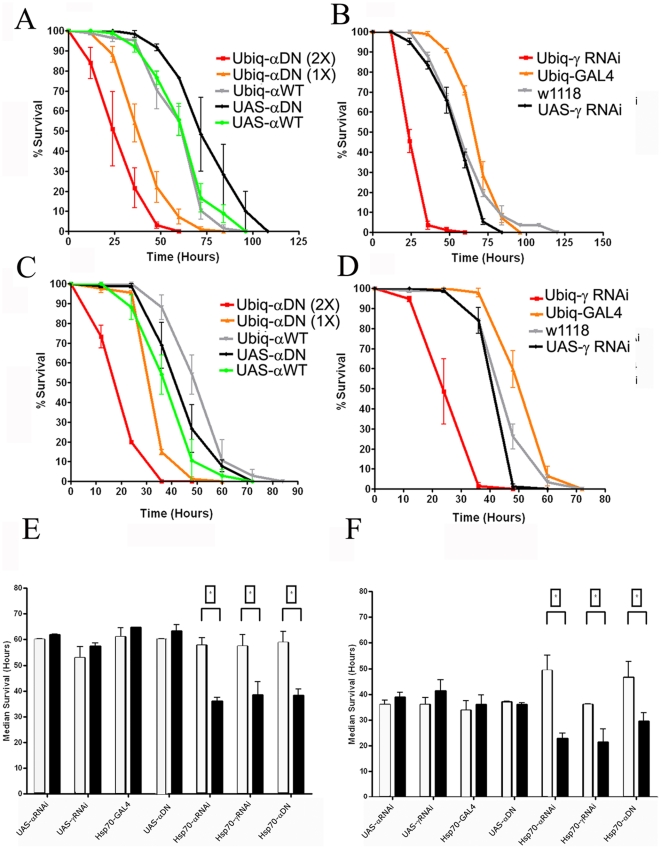
Reduction of AMPK function causes a reduced lifespan under starvation. Survival curves from adult females (A) and males (C) expressing either one or two copies of the AMPK^DN^ transgenes under the control of the Ubiq-GAL4 driver. Expression of these transgenes reduced lifespan compared to the parental controls or animals expressing a wild-type α subunit. Survival curves of adult females (B) and males (D) expressing an RNAi element targeting the gamma subunit. The lifespan of these animals was significantly shorter than the parental lines or the *w^1118^* genetic background control (p<0.05, ANOVA). (E) Female and (F) male adult induction of the AMPK-interfering transgenes significantly reduces median lifespan (* indicate significant differences between non-induced (unfilled bars) and induced (filled bars) median survival (p<0.05, two-tailed T-test)).

Having validated that these genetic strategies were producing phenotypes consistent with reduced AMPK activity, we then asked whether attenuated AMPK function would alter sensitivity to metabolic challenges. We tested the progeny of a UAS-AMPK^DN^ under the control of a Ubiquitin-GAL4 (Ubiq) driver for adult survival during starvation, as this driver recapitulates the global expression pattern of the ubiquitin gene, but is not as strong as the Act5C-GAL4 driver [22]. We found a significant reduction of lifespan in animals expressing either the dominant negative alpha transgene (Figure 2A, C) or the gamma RNAi element (Figure 2B, D) as compared to animals expressing a wild-type alpha sequence or to parental stocks. As expected, there was a strong impact of dosage of the alpha dominant negative transgene. Notably, expression of a wild-type copy of the alpha subunit in conjunction with the dominant negative element attenuated starvation sensitivity phenotypes (Figure S4), providing genetic evidence that the K57A variant possesses dominant negative action.

Given the developmental defects exhibited in animals lacking functional AMPK, we performed experiments to assess the extent to which potential developmental defects caused by impaired AMPK function contribute to starvation sensitivity in adults. We therefore employed the hsp70-GAL4 element, which allows temporal control through stage-specific induction by heat-shock and thus avoid any developmental disturbances caused by earlier transgene expression. Heat-shock-induced AMPK impairment in adult stages produced a significant reduction in starvation survival (Figure 2E-F), and starvation sensitivity in animals bearing AMPK transgenes were indistinguishable from wild-type animals under uninduced experimental conditions. We also assessed aging in these animals and found a significant reduction in lifespan under nutrient-rich conditions (Figure S5), although reduced AMPK function did not significantly impact lifespan for younger cohorts used for starvation studies.

### Reduced AMPK causes changes in locomotor activity

**Figure 3:**
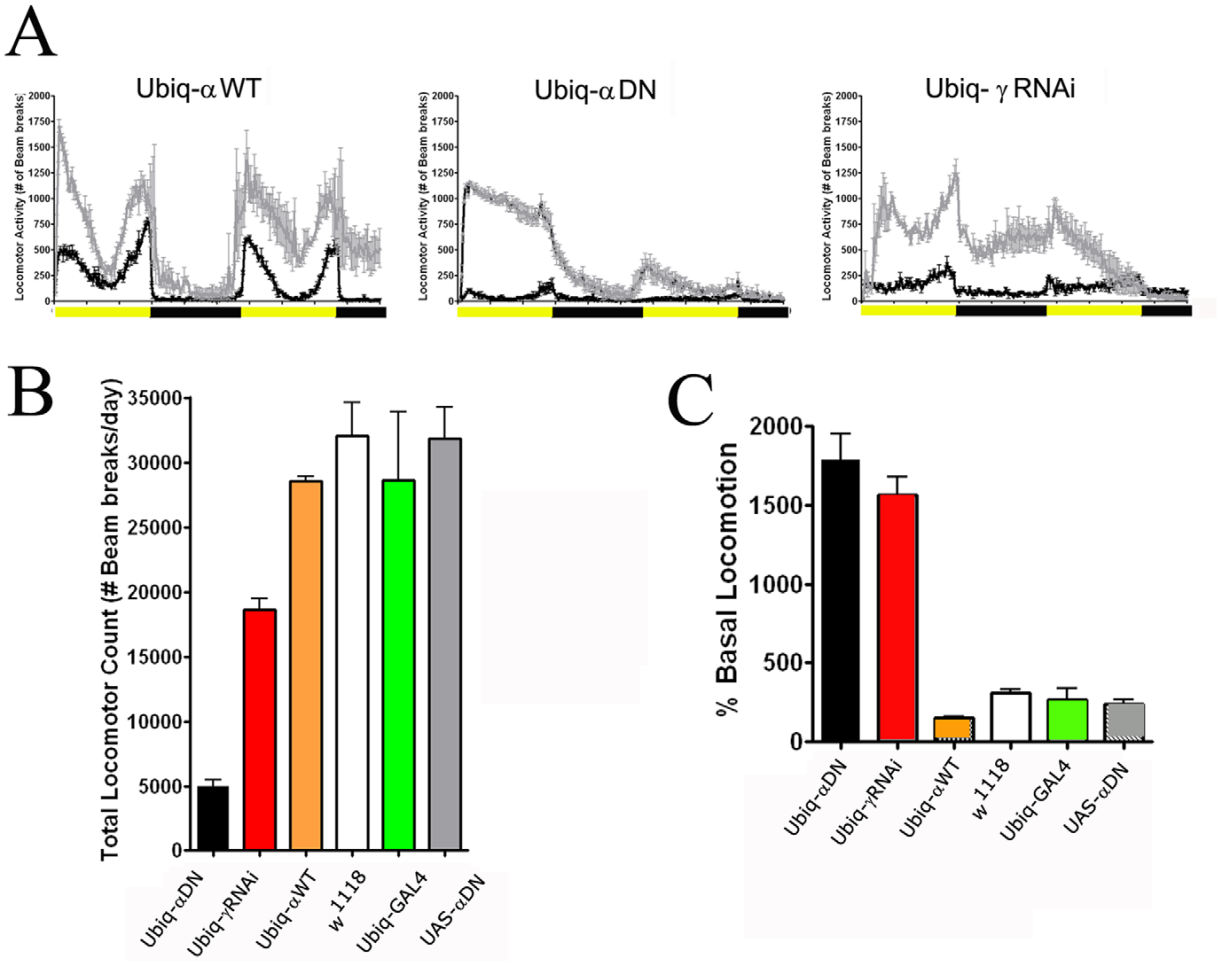
Locomotor activity and starvation-induced hyperactivity is altered as a consequence of reduced AMPK signaling. Locomotor activity measured during fed (black line) and starved conditions (gray line) in female animals (A) either expressing the wild-type α subunit (left), the dominant negative α transgene (middle), and the γ RNAi element (right) for forty-eight hours. Quantification of total locomotion during fed conditions (B) and the magnitude of starvation-induced hyperactivity (C). Measurements of locomotor activity were initiated at ZT0 (lights on) and flies were maintained in a 12∶12 LD (light-dark) cycle (shaded and dark bars). (B) Expression of either the α dominant negative or the γ RNAi element lead to reduced locomotor activity during the fed state (ANOVA, P<0.05). (C) Both the γ RNAi element and the α dominant negative variant caused greater levels of starvation-induced activity relative to basal locomotion (ANOVA, P<0.05).

Starvation induces hyperactivity in *Drosophila* and other Metazoa, which is believed to be an adaptive response, as elevated activity promotes increased foraging [23]–[24]. Given the profound nature of the sensitivity to starvation in animals with reduced AMPK function, we hypothesized that a potential mechanism of such sensitivity may be a greater level of hyperactivity. The rationale is that accelerated activity would have the net effect of a more rapid exhaustion of energy stores. We first evaluated normal locomotor activity levels in animals with reduced and wild-type AMPK. Animals expressing the dominant negative AMPK α transgene or γ RNAi had significantly lower amounts of locomotion during normal, unstressed conditions as compared to animals expressing a wild-type AMPK α subunit or to *w^1118^* genetic background controls (Figure 3A) (ANOVA – Tukey posthoc comparison P<.001). However, while wild-type animals show a steady increase in activity levels as a function of starvation duration, animals expressing the dominant negative AMPK transgene or the gamma RNAi element displayed instantaneous elevated activity levels (Figure 3B, C). Notably, this pattern of locomotor activity was not evident in any of the parental lines.

### Reduced AMPK causes hyperphagia

**Figure 4:**
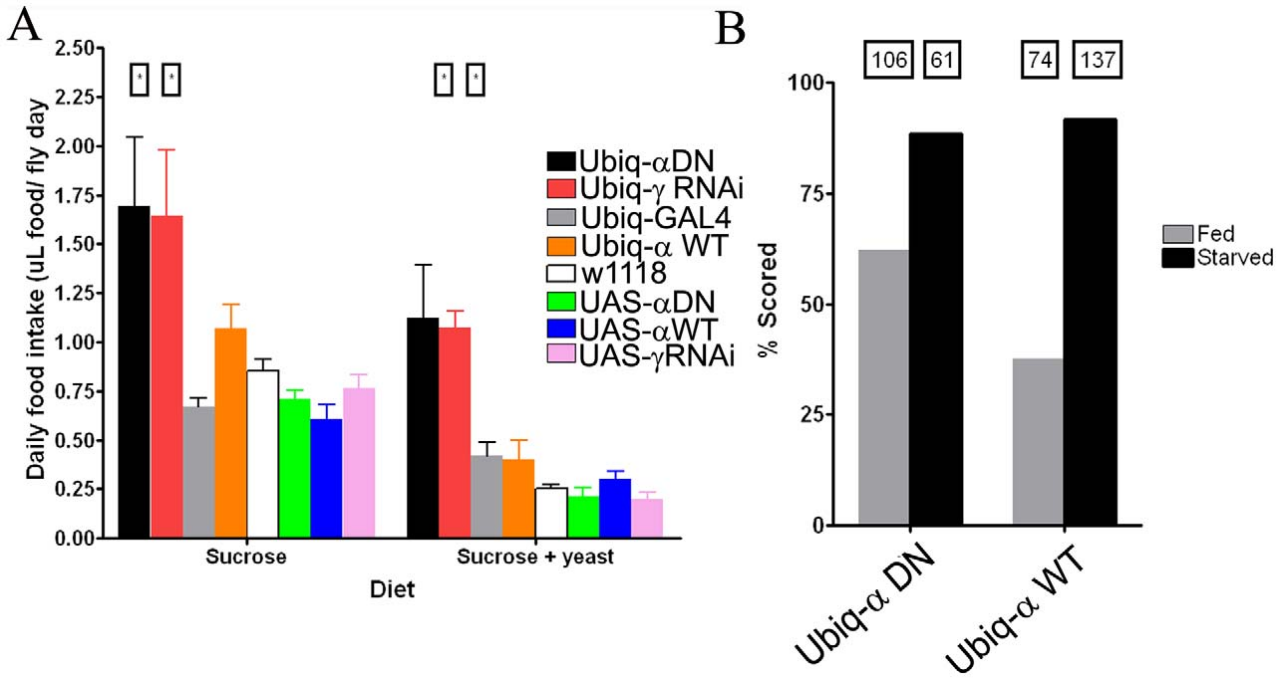
Altered feeding behaviors in animals with decreased AMPK function. (A) Daily food intake in adult *Drosophila* with different AMPK functional levels with a sucrose diet (left) or a yeast-sucrose diet (right). Males and females showed no difference in food intake and data was pooled. Six trials consisting of three trials each were measured for one day. Asterisks denote statistical significance (ANOVA, p<0.05) compared to all other genetic controls. (B) Abdominal scoring in animals expressing either a wild-type or the dominant negative alpha subunit following either 12 hours of fed conditions (grey bars) or starvation (black bars). Numbers in boxes represent the total number of flies scored for a particular genotype. Animals were given two hours of exposure to the food with the dye and then anaesthetized and abdomens were scored for blue-dye. A G-test for statistical significance showed no differences in scoring of animals between genotypes under prior starvation conditions (p>0.05), whereas the amount of individuals actively feeding following the fed conditions were different between genotypes (p<0.01).

Since the loss of AMPK function causes exaggerated responses to starvation, we speculated that this may be caused by reduced food intake. Further rationale comes from mammalian studies where AMPK activity has been shown to regulate the expression of orexigenic transmitters [25]. To test this hypothesis, we measured total dietary intake employing the CAFE assay [14]. Total daily food intake was significantly increased in animals expressing the AMPK^DN^ or AMPK gamma RNAi variant and this increase was evident, independent of nutritional value (Figure 4A). Specifically, in both males and females, total dietary intake was nearly twice that of animals with wild-type AMPK function (P<0.0001, F = 29.09, Two-Way ANOVA). This implicates that not only are animals with reduced AMPK function actively feeding, but that they also display hyperphagia. This hyperphagia might be caused by a persistent starvation signal/phenotype. To test whether loss of AMPK leads to altered hunger-driven feeding behavior, we scored individuals for incorporation of blue dyed food in the abdomens. A variation of this assay was originally developed to assess whether compounds were aversive or appetitive, and require a period of starvation to achieve significant numbers of individuals that feed enough to facilitate scoring [15]. We reasoned that if animals deficient in AMPK were consistently starved, then higher rates of scored individuals would be attained without prior starvation conditions. Consistent with these predictions, we found that animals expressing the dominant negative AMPK construct incorporated the dye at a higher level (62.2%) compared to animals with wild-type AMPK function (37.7%). Notably, comparable numbers of individuals were scored following a period of starvation, independent of genotype (88.5% for AMPK^DN^ and 91.8% for AMPK^WT^) (Figure 4B).

### Abnormal lipid accumulation phenotypes caused by altered AMPK

**Figure 5:**
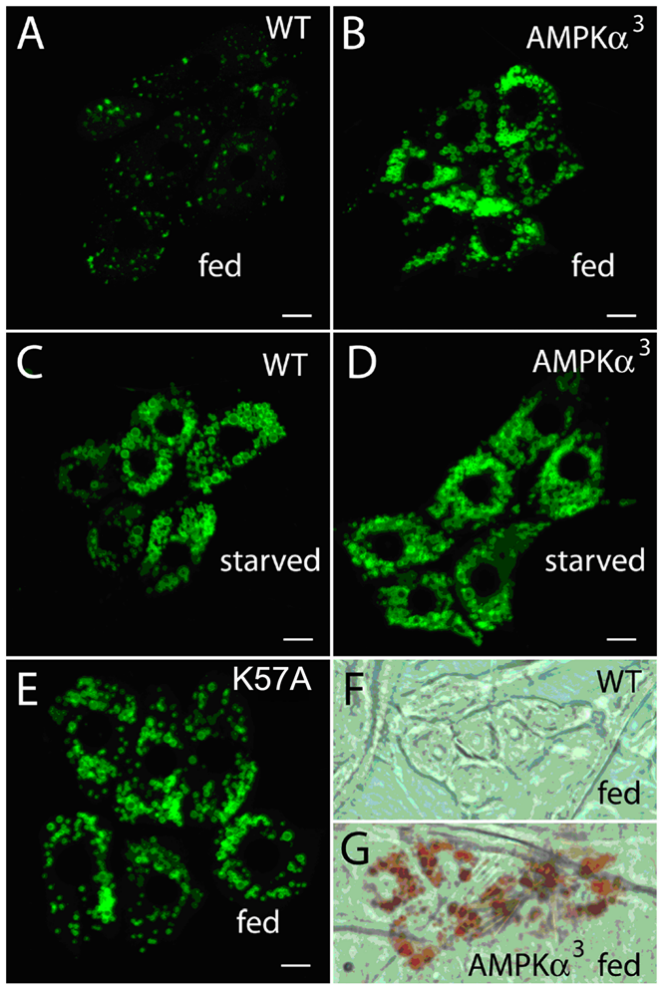
Abnormal lipid accumulation in oenocytes in animals with reduced AMPK. Oenocytes in the larva are a specialized cell type that functions similar to mammalian liver and accumulates lipids during starvation (13). We introduced a LSD2-GFP fusion to oenocytes to observe the size and quantity of lipids in oenocytes in genetic backgrounds differing in AMPK function. In wild-type animals under fed conditions, the LSD2-GFP labeled lipid droplets were small and not prominent (A), whereas in the starved state, both the size and number of lipid droplets increase (C). In contrast, in AMPKα deficient larvae, there was no difference between the fed and starved states (B and D) and these closely resemble the starved state of wild-type animals. This phenotype was also exhibited in animals expressing the dominant negative AMPK subunit in oenocytes (E). Likewise, the amount of Oil-O red staining was notably absent in wild-type animals during fed conditions (F) but labeled significantly more droplets in animals lacking AMPK function during the fed state (G).

Animals with reduced AMPK function demonstrate hypersensitivity to starvation, despite also displaying hyperphagia, suggesting that these animals are persistently experiencing starvation. Because AMPK null mutants die as larvae and that these larvae are characteristically smaller than wild-type larvae of the same age (J.E. Brenman, personal observations), we speculated that persistent starvation may be the ultimate cause of reduced larval growth and lethality. We took advantage of previous observations that starvation induces an accumulation of lipid droplets in larval oenocytes [13], and consequently, we examined whether oenocytes from animals with reduced AMPK would show aberrant oenocyte lipid accumulation phenotypes. We introduced a lipid marker: LSD2-GFP, [26] in larvae with either wild-type AMPK function or lacking AMPK alpha gene function [9]. Under fed conditions, animals with wild-type AMPK function showed little to no lipid droplets present in oenocytes as expected and previously published [13], whereas in contrast, larvae lacking AMPK showed significantly more droplets and larger droplets per cell, resembling the starved phenotype of wild-type oenocytes (Figure 5). This phenotype was also evaluated using Oil-O-red staining which independently labels lipid droplets and circumvents over-expression of the LSD2-GFP marker, yet similar patterns were still observed (Figure 5A-G), specifically, that oenocytes with reduced AMPK function show significant lipid accumulation in the fed state.

### Altered metabolism in animals with reduced AMPK function

**Figure 6:**
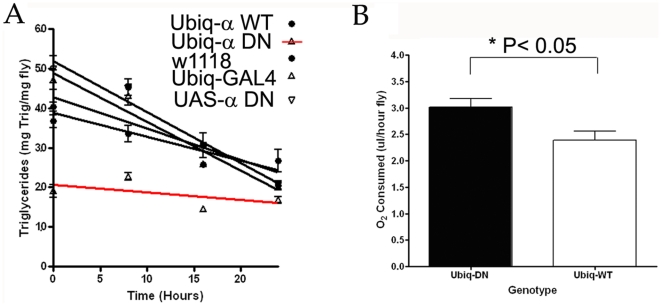
Altered metabolism in animals with reduced AMPK function. We quantified total triglyceride levels in adult females with different levels of AMPK function (A). Animals expressing the dominant negative construct under the control of the Ubiq-GAL4 driver, show significantly lower triglyceride levels during fed conditions (Time 0). We also tested oxygen consumption in animals expressing either the dominant negative or a wild-type alpha subunit (B). Animals expressing the dominant negative have higher O_2_ consumption than animals expressing a wild-type alpha subunit.

Given these observations of persistent starvation phenotypes, we then next tested to see if the size of stored energy reserves were impacted in animals with attenuated AMPK function. We quantified triglyceride levels in animals with either mutant or wild-type AMPK function during normal and starvation conditions. While there were no overt differences in weight, there was a significant impact of the AMPK^DN^ on total triglyceride stores under fed conditions; with the average amount of triglyceride levels for the dominant negative being significantly lower than AMPK-wild type expressing flies (Figure 6A). This result suggests a specific effect of AMPK on lipid amount and that the decreased amounts of triglyceride stores may cause abbreviated lifespan during starvation in animals with reduced AMPK function.

While this observation might explain shortened survival under energetic stress, lower triglyceride levels resulting from a loss of AMPK function contradicts previous reports that activation of AMPK causes reduced triglyceride turnover [27]. However, this result is consistent with a previous observation in *C. elegans* demonstrating that AMPK mutants do not properly utilize lipid stores during energetic stress [28]. In attempts to reconcile these observations, we hypothesized that reduced triglyceride levels concomitant with hyperphagia may be caused by increased metabolic demand present in animals with reduced AMPK function. If this were the case, then we reasoned that these animals might exhibit higher consumption of oxygen. Measurements of O_2_ consumption in animals expressing the dominant negative alpha construct were consistently higher than in animals expressing a wild-type alpha subunit (P<0.0002, F = 47.07, Repeated Measures ANOVA) (Figure 6B).

### Rapamycin alleviates AMPK phenotypes

**Figure 7:**
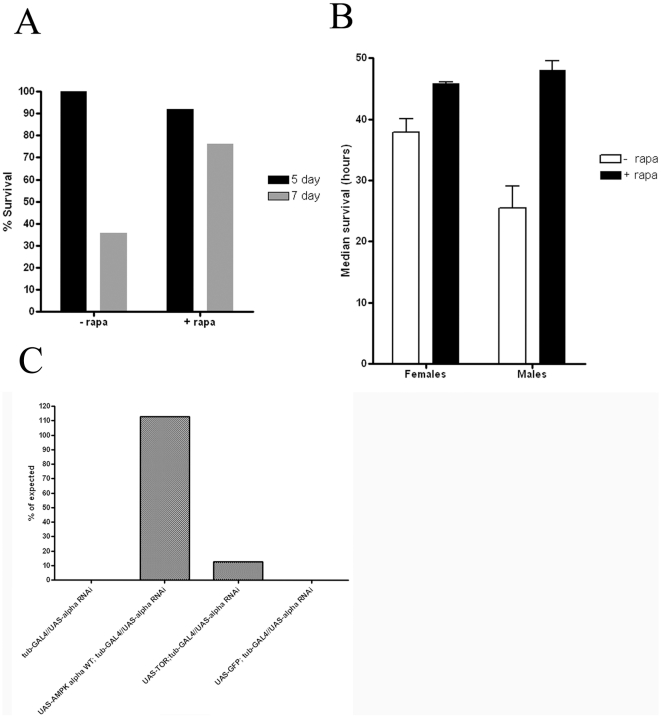
Rapamycin increases survival rates caused by reduced AMPK function. (A) We fed *AMPK*α mutant larvae rapamycin and evaluated survival. The majority of the lethal effects of the *AMPK*α mutation occur during the 5^th^ and 7^th^ day of the third instar. Incorporation of rapamycin reduced this lethality during this timeframe. (B) Likewise, rapamycin feeding for three days prior to starvation improved survival of adult animals expressing the dominant negative variant. (C) Introduction of a TOR dominant negative partially rescues lethality associated with the alpha RNAi.

Given the observations that animals with reduced AMPK function have higher metabolic rates, we tested the hypothesis that this metabolic activity may stem from failures to reallocate energy during nutritional stress. AMPK signaling can intersect with the target of rapamycin (TOR); AMPK activation would lead to TOR inhibition, which would lead to decreased protein synthesis and decreased energy consumption. [29]. We fed rapamycin, a TOR inhibitor, to larvae possessing the null allele of the α subunit and tested if this agent impacted the lethality associated with this mutation. We identified that the majority of AMPK deficient larvae died between the fifth and seventh day of the third instar, but that rapamycin increased the percentage of mutant larvae surviving to the seventh day (Figure 7A). We next tested whether rapamycin would likewise improve the starvation sensitivity in adult animals with reduced AMPK function. The median survival of these animals significantly improved during starvation conditions for females (P = 0.003 T-Test) and males (P = 0.0005 T-Test) (Figure 7B). Furthermore, expression of a dominant negative form of TOR [30]–[31], partially rescued the lethality caused by global expression of the alpha RNAi element (Figure 7C).

## Discussion

We report that animals with reduced AMPK function are highly susceptible to the effects of starvation, and note significant changes in behavioral and physiological responses to starvation caused by altered AMPK function. Demonstrations of increased hyperactive locomotor responses, lower triglyceride levels, increased hunger-driven feeding behaviors, higher metabolic rates, and lipid accumulation phenotypes in liver-like oenocytes indicate that the loss of AMPK leads to a persistent starvation signal both at the organismal and cellular levels.

These phenotypes suggest mechanisms underlying reduced survival during starvation in animals with altered AMPK activity. For example, starvation induces hyperactivity in flies with wild-type AMPK function [23]–[24], and we observed hyperactive responses which occurred more rapidly in animals with reduced AMPK function. Larger hyperactive responses require more energy and under limiting conditions, would lead to compromised abilities to withstand nutritional stress. Likewise, reduced triglyceride storage would effectively limit the ability to fuel such energy-requiring responses. Lastly, oenocytes are thought to be involved in the processing of fats making them ready for utilization [13], suggesting that the accumulation phenotypes seen here may be linked to the observed lower global triglyceride levels.

We suspect that many of these starvation phenotypes are consequences of a core metabolic defect. While we cannot rule out potential pleitropic effects, we suggest our results are consistent with an idea of compensatory changes in critical physiologies and behaviors stemming from defects in energy allocation, storage, or utilization. Our observations that rapamycin significantly extends lifespan, and that animals with reduced AMPK function consume more oxygen indicate inabilities to appropriately regulate cellular metabolic activities. Our observations are also consistent with multiple observations that AMPK is an important factor regulating cellular activities, and regulate expenditures of energy including protein synthesis [32]. Furthermore, similar observations have been made regarding AMPK function in the nematode, *Caenorhabditis elegans*. Upon exposure to harsh environmental conditions, including nutrient limitation, *C. elegans* enter a dormant dauer state to become stress-resistant. *C. elegans* larvae lacking AMPK activity rapidly consume their energetic stores and die prematurely and are unable to utilize energy efficiently [28]. In addition, altered locomotor activity, lower triglycerides, and higher oxygen consumption rates are also caused by a loss of AMPK function in this genetic model organism [28], [33], which parallels our findings in *Drosophila*. In mammals, a recent report of altered AMPK activity in the mouse also shows some similar phenotypes, specifically higher locomotor activity and reduced triglyceride levels [21]. While AMPK has been shown to cause neurodegeneration [34]–[35], we suspect that the loss of neural centers as a consequence of attenuated AMPK function is not responsible for the behavioral phenotypes, as the timecourse of neurodegeneration is inconsistent with the acute behavioral responses we observed. Likewise, pan-neuronal expression of an RNAi element in *Drosophila* does not alter starvation sensitivity [36], further suggesting that these effects are most likely a specific consequence of AMPK in metabolic pathways as opposed to AMPK impact on neuron viability.

While such observations are consistent with the suspected roles of AMPK as a regulator of cellular activities, our results with an organismal reduction of AMPK function are not entirely expected based on all previous studies and predictions of AMPK function. For example, AMPK is involved in the release of orexigenic peptides from the mammalian hypothalamus during starvation conditions, which acts to stimulate feeding during low energy [25]. Unexpectedly, our results with *Drosophila* with compromised AMPK function globally show a similar hyperphagic response. Additionally, AMPK has been shown to inhibit lipolysis [37], yet our results show that reduced AMPK function leads to lower triglyceride levels instead of higher levels. We maintain that our results do not specifically conflict with these reported roles for AMPK. We note that starvation induces a series of integrated cell-autonomous and hormonal mechanisms to coordinate organismal changes in behavior and physiology, with different cellular populations responding differently [38]. Therefore, AMPK is not the singular sensor for low energy levels, and indeed other mechanisms of restoring energy (*i.e*., autophagy), which can be independent of AMPK [39], are presumably responsible for modified behavioral phenotypes in our whole animal reduction of AMPK function. Likewise, we suspect that in some tissues the loss of AMPK may lead to resistance to nutrient deprivations, through the critical loss of behaviors and/or physiologies that would exhaust energy stores rapidly. We also note that the transgene-drivers employed here may not reduce AMPK levels in all cellular populations equally, although we note that two global drivers (Ubiq-GAL4 and hsp70-GAL4) do give similar phenotypes. Thus, our results may provide insight into the identification of other factor(s) that are likewise involved in mediating metabolic homeostasis.

We also note that these phenotypes likely stem from the acute loss of AMPK function as opposed to developmental defects caused by reduced AMPK signaling. While AMPK is clearly required for normal development, as null mutants are larval lethal [9], we note that induced transgene expression in adults leads to reduced survivorship during starvation. We suspect that the metabolic phenotypes associated with attenuated AMPK signaling are also the cause of the lethality associated with the loss of AMPK function, as repression of TOR provided some rescue of lethality associated with the loss of AMPK function. These larvae are slow-growing, despite actively feeding. Our observations of abnormal lipid accumulation by larval oenocytes demonstrate that the loss of AMPK also leads to persistent starvation conditions [13] at least by some cell types.

Our results suggest a series of future experiments targeting AMPK function in various central populations that may mediate feeding behaviors, such as neurons that express neuropeptide F or the *hugin* – encoding peptides, both of which have been previously shown to modulate larval feeding [40]–[41]. Likewise, other potential cell-specific roles of AMPK are now open to investigation in *Drosophila*, including muscle function, liver lipolysis, and locomotor behaviors. For example, the neuroendocrine cells that produce the Adipokinetic Hormone (AKH) are critical for the formation of starvation-induced hyperactive behaviors [19]–[20]. AMPK may participate in this specialized population of cells to regulate AKH signaling, as well as in other potential cells including insulin [42], which is known to participate in the endocrine events that shape behavioral and physiological responses to starvation. During preparation of this manuscript, two independent reports showed similar phenotypes stemming from reduced AMPK function [36], [43]. One report showed a similar sensitivity to starvation conditions as we have, in *Drosophila* with reduced AMPK function through selective introduction of an RNAi element targeting the alpha subunit. Moreover, this report suggests that the selective loss of AMPK signaling in muscle tissue leads to heightened sensitivity to starvation [35]. Another study show a reduction of triglycerides in larval stages in animals bearing a null mutation in the alpha subunit, and reports that gut expression of AMPK is critical for this early developmental lethality [36]. Again, we suspect multiple tissues to show variable phenotypes; this suspicion is based on the multiple functional roles reported for the AMPK molecule, and we have begun assessing starvation phenotypes in discrete tissues in *Drosophila*.

In addition, if AMPK functions noted here in *Drosophila* and previously in *C. elegans* [13], [44] are conserved in humans, some thought to global activation of AMPK as a therapeutic should be considered. Observations in *Drosophila* (here) and *C. elegans* suggest loss of AMPK activity leads to difficulty maintaining energy stores and inefficient utilization of them; in essence excessive use of energy stores. If the opposite were true during AMPK activation (for instance with an AMPK-activating drug in humans) one might predict highly-efficient and sparing utilization of energy stores (fat). Since Type 2 Diabetes is associated with obesity and a sedentary lifestyle, such an effect of AMPK activators would be very undesirable and potentially lead to increased energy stores (fat).

## Supporting Information

Figure S1 Ubiquitous expression of AMPK alpha K57A and AMPK gamma RNAi elements cause lethality. Adult animals of specific genotypes were counted, as determined by the presence or absence of specific markers. Percent of expected was calculated as determined by expected Mendelian segregation ratios (assuming no survival effect of any transgene). Numbers above the columns refer to the total number of animals scored. Note that both RNAi elements and the K57A (DN: dominant negative) phenocopy the lethality caused by the AMPKα mutation and is dependent upon GAL4 mediated transcription.
(1.11 MB TIF)

Figure S2 Failure of the K57A transgene to rescue lethality caused by the molecular null ampk allele. We evaluated the extent to which different AMPK transgenes were able to rescue the lethality caused either by the AMPK3 mutation or by expression of the K57A element. Animals were scored with respect to segregation of dominant markers present on balancer chromosomes.
(3.70 MB TIF)

Figure S3 Reduced expression of AMPKα protein caused by expression of different RNAi elements. (A) Western blot for the AMPKα subunit from lysates of animals expressing RNAi for either the alpha (second lane) or the gamma subunit (third lane). Compared to animals possessing the driver alone (first lane) or an RNAi targeting an unrelated gene (ATG8) (fourth lane), there was significantly less expression of the alpha subunit standardized to loading control (tubulin). (B) Semi-quantitative RT-PCR of gamma expression (left) and RP49 (right) derived from animals expressing the gamma RNAi element compared to wild type. Under these PCR conditions, there was no detectable band for the gamma transcript in animals expressing the specific RNAi element.
(1.81 MB TIF)

Figure S4 Median survival time during starvation for animals with reduced AMPK function is decreased. Median survival was determined from three replicate vials of thirty individuals. Non-linear regression was employed to estimate a median survival for each vial; data were pooled, and mean ±SEM is plotted. The addition of a wild-type copy to animals expressing the K57A element leads to intermediate starvation survival times, implicating the K57A as a dominant negative.
(5.15 MB TIF)

Figure S5 Aging is significantly impacted by altered AMPK function. We measured percent survival daily from 300 males and 300 females either expressing the dominant negative or wild type subunit under normal fed conditions, once daily. A non-linear regression was employed to estimate median survival for each vial, and a mean ±SEM is plotted. Overexpression of the wild type alpha subunit leads to increased longevity as compared to w1118 (the genetic background), and in contrast, expression of the dominant negative element leads to decreased longevity (asterisks indicate statistically significance from w1118 [P<0.001, Two-Way ANOVA]).
(3.16 MB TIF)
