# Supplementary figures and images for: Altered Metabolism and Persistent Starvation Behaviors Caused by Reduced AMPK Function in Drosophila
Source: PLoS One. 2010 Sep 20;5(9):e12799. doi: 10.1371/journal.pone.0012799 (PMC2942814; doi:10.1371/journal.pone.0012799)

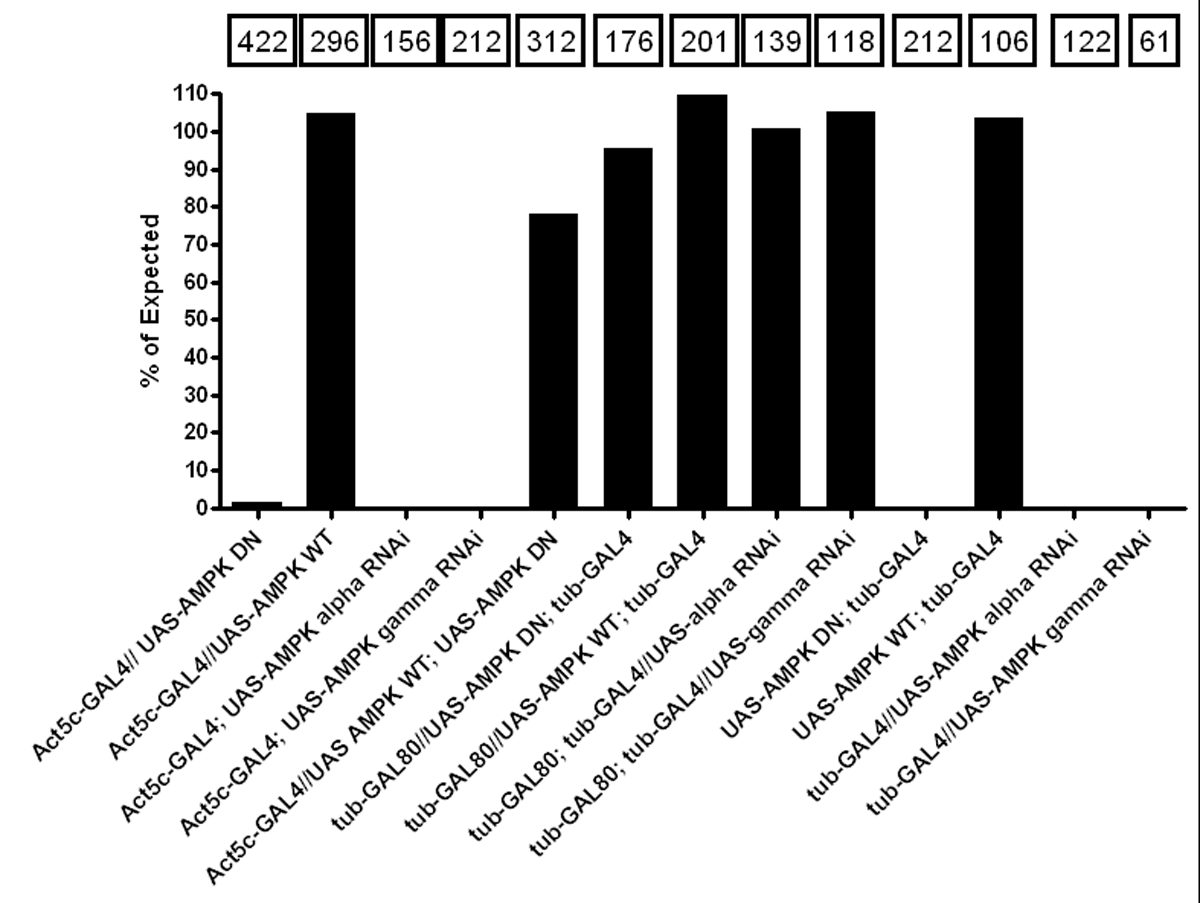

Supplement: Figure S1 — Ubiquitous expression of AMPK alpha K57A and AMPK gamma RNAi elements cause lethality. Adult animals of specific genotypes were counted, as determined by the presence or absence of specific markers. Percent of expected was calculated as determined by expected Mendelian segregation ratios (assuming no survival effect of any transgene). Numbers above the columns refer to the total number of animals scored. Note that both RNAi elements and the K57A (DN: dominant negative) phenocopy the lethality caused by the AMPKα mutation and is dependent upon GAL4 mediated transcription. (1.11 MB TIF) [file pone.0012799.s001.tif]

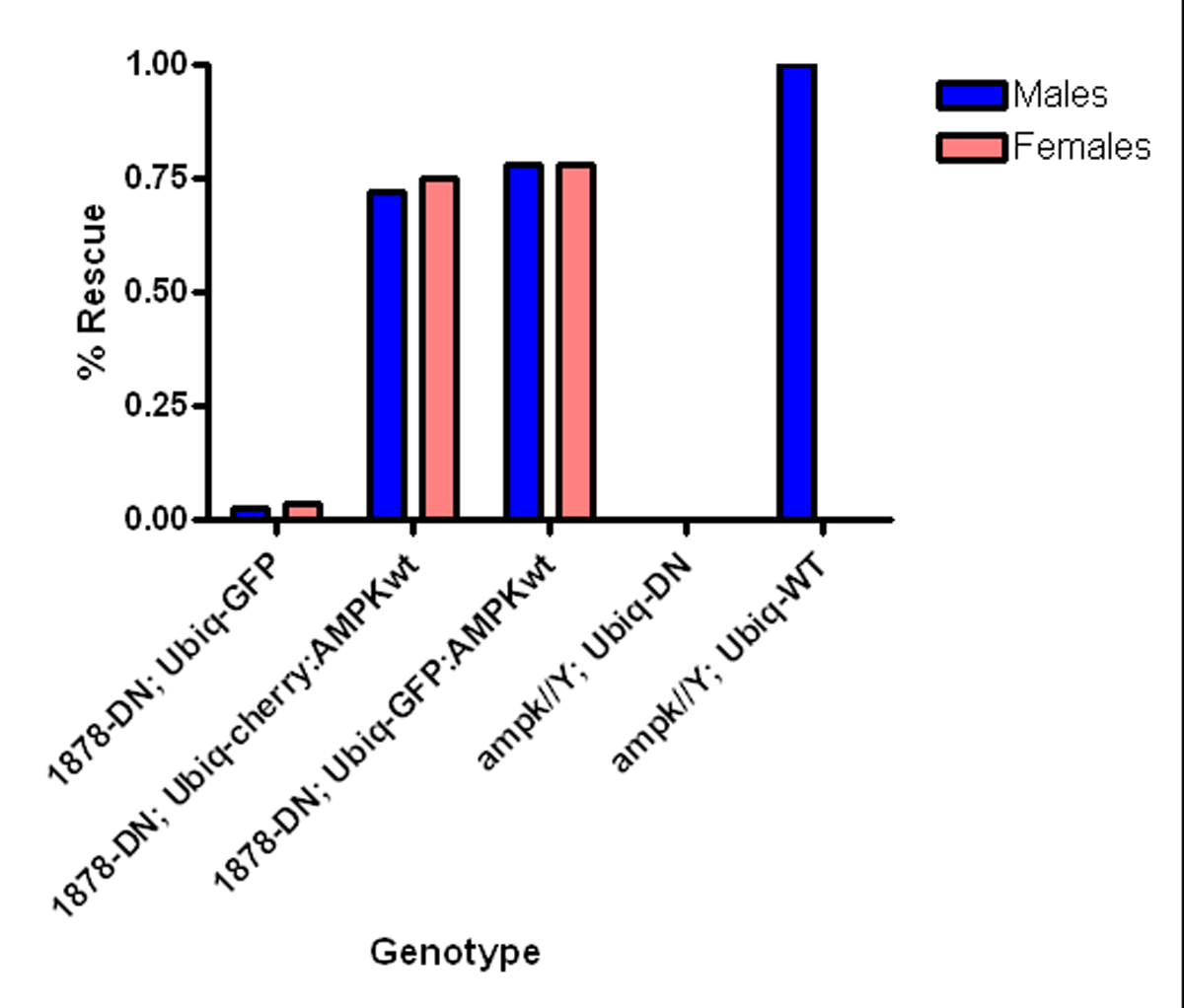

Supplement: Figure S2 — Failure of the K57A transgene to rescue lethality caused by the molecular null ampk allele. We evaluated the extent to which different AMPK transgenes were able to rescue the lethality caused either by the AMPK3 mutation or by expression of the K57A element. Animals were scored with respect to segregation of dominant markers present on balancer chromosomes. (3.70 MB TIF) [file pone.0012799.s002.tif]

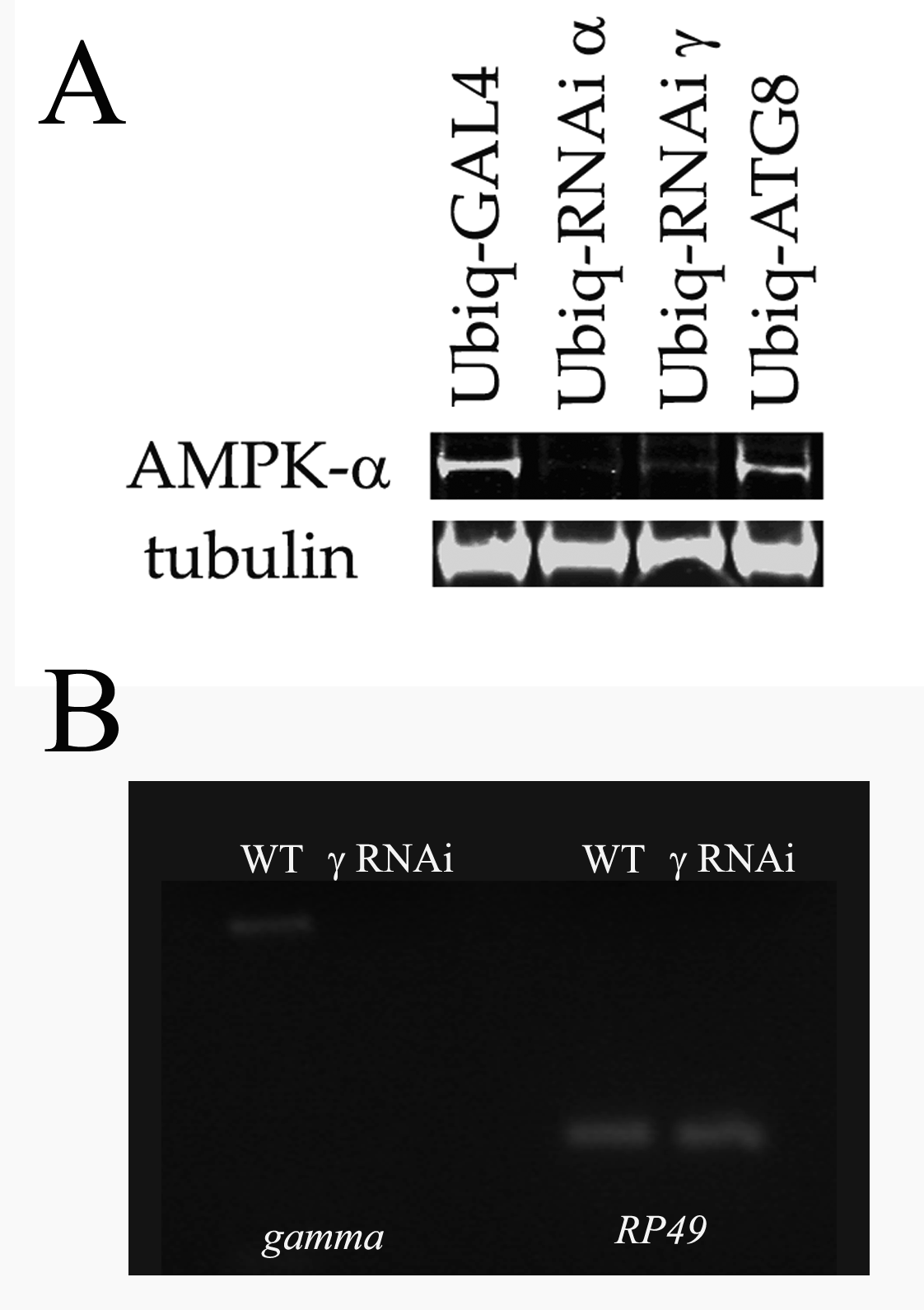

Supplement: Figure S3 — Reduced expression of AMPKα protein caused by expression of different RNAi elements. (A) Western blot for the AMPKα subunit from lysates of animals expressing RNAi for either the alpha (second lane) or the gamma subunit (third lane). Compared to animals possessing the driver alone (first lane) or an RNAi targeting an unrelated gene (ATG8) (fourth lane), there was significantly less expression of the alpha subunit standardized to loading control (tubulin). (B) Semi-quantitative RT-PCR of gamma expression (left) and RP49 (right) derived from animals expressing the gamma RNAi element compared to wild type. Under these PCR conditions, there was no detectable band for the gamma transcript in animals expressing the specific RNAi element. (1.81 MB TIF) [file pone.0012799.s003.tif]

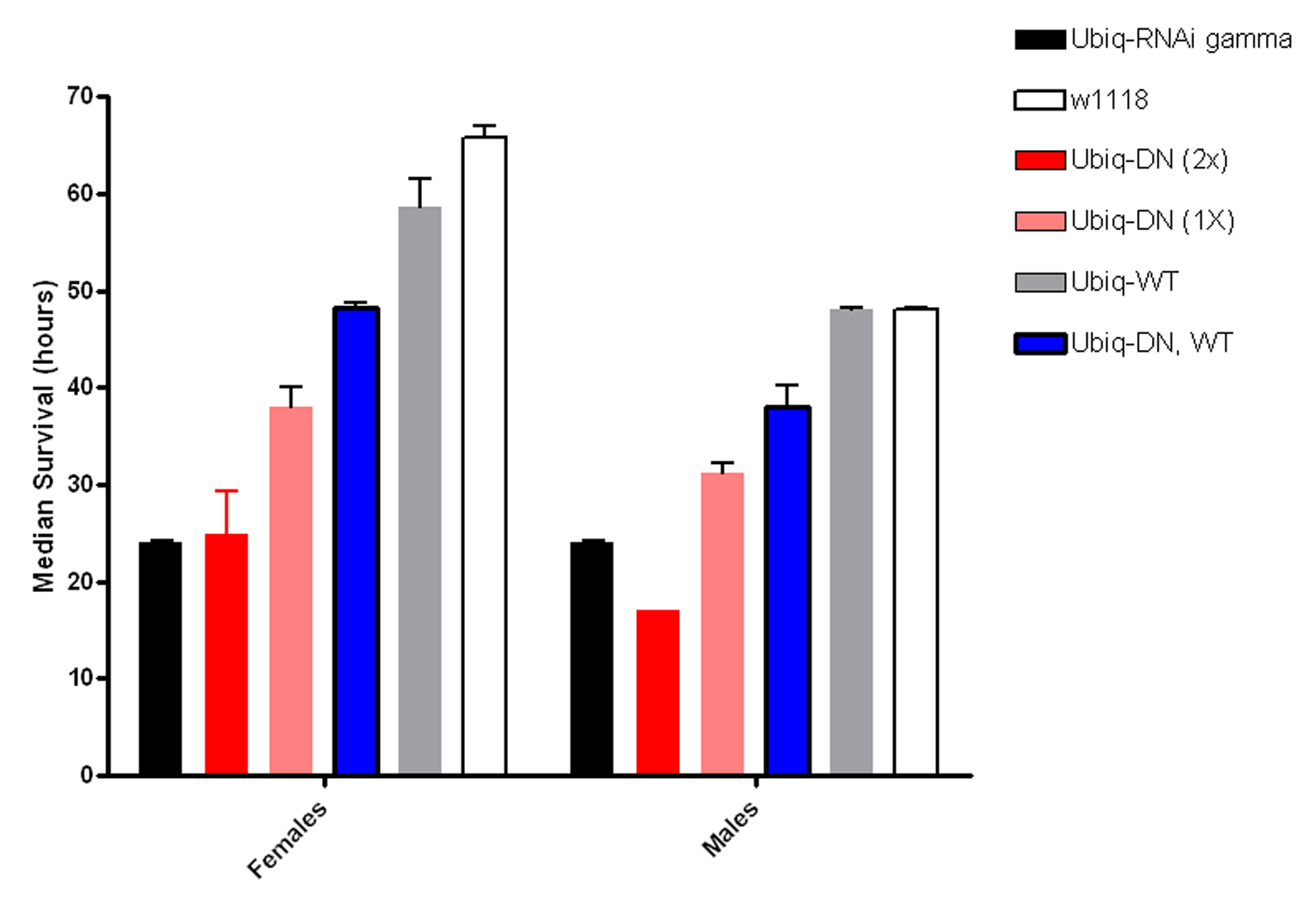

Supplement: Figure S4 — Median survival time during starvation for animals with reduced AMPK function is decreased. Median survival was determined from three replicate vials of thirty individuals. Non-linear regression was employed to estimate a median survival for each vial; data were pooled, and mean ±SEM is plotted. The addition of a wild-type copy to animals expressing the K57A element leads to intermediate starvation survival times, implicating the K57A as a dominant negative. (5.15 MB TIF) [file pone.0012799.s004.tif]

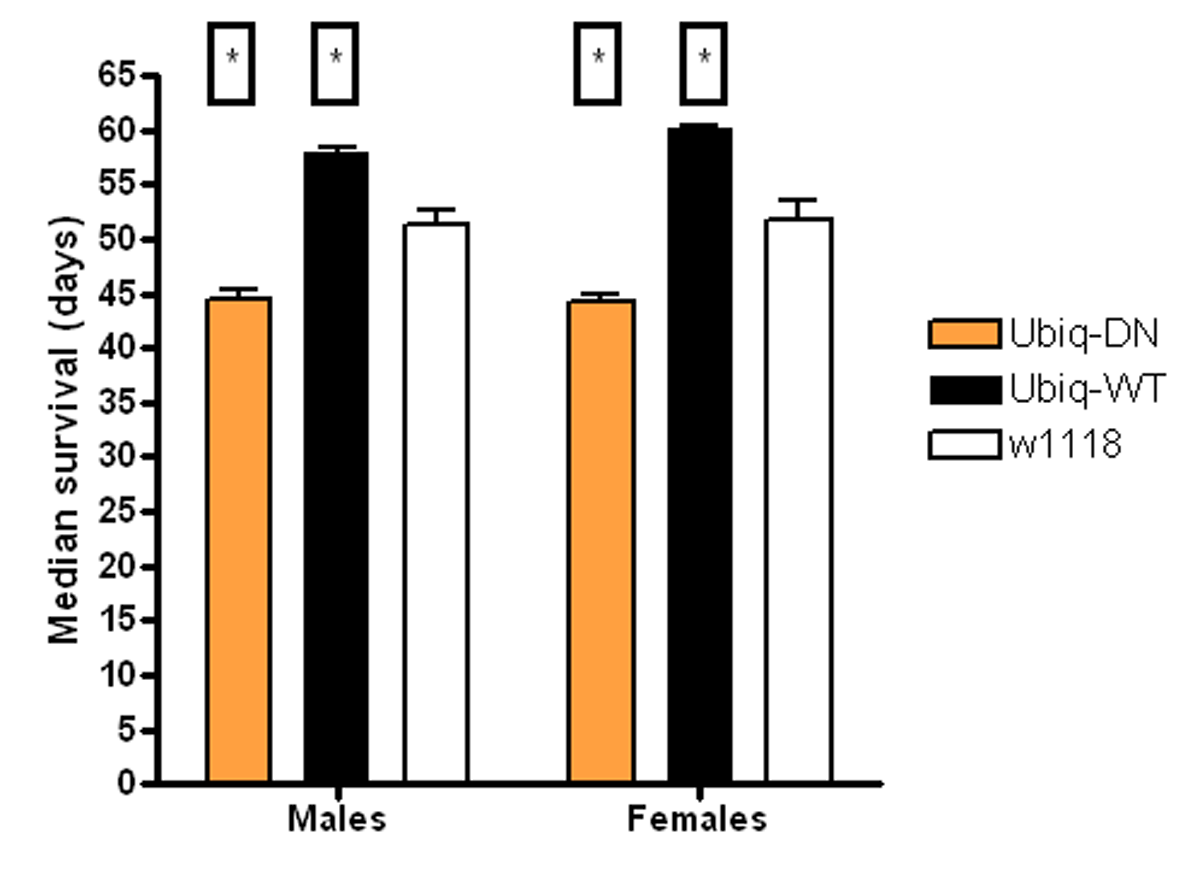

Supplement: Figure S5 — Aging is significantly impacted by altered AMPK function. We measured percent survival daily from 300 males and 300 females either expressing the dominant negative or wild type subunit under normal fed conditions, once daily. A non-linear regression was employed to estimate median survival for each vial, and a mean ±SEM is plotted. Overexpression of the wild type alpha subunit leads to increased longevity as compared to w1118 (the genetic background), and in contrast, expression of the dominant negative element leads to decreased longevity (asterisks indicate statistically significance from w1118 [P<0.001, Two-Way ANOVA]). (3.16 MB TIF) [file pone.0012799.s005.tif]
